# Senescence and Apoptosis During *in vitro* Embryo Development in a Bovine Model

**DOI:** 10.3389/fcell.2020.619902

**Published:** 2020-12-18

**Authors:** Priscila Ramos-Ibeas, Isabel Gimeno, Karina Cañón-Beltrán, Alfonso Gutiérrez-Adán, Dimitrios Rizos, Enrique Gómez

**Affiliations:** ^1^Department of Animal Reproduction, National Institute for Agriculture and Food Research and Technology (INIA), Madrid, Spain; ^2^Servicio Regional de Investigación y Desarrollo Agroalimentario (SERIDA), Gijón, Spain

**Keywords:** assisted reproductive technologies, programmed cell death, cell cycle arrest, DNA fragmentation, TUNEL, caspase

## Abstract

According to the World Health Organization, infertility affects up to 14% of couples under reproductive age, leading to an exponential rise in the use of assisted reproduction as a route for conceiving a baby. In the same way, thousands of embryos are produced in cattle and other farm animals annually, leading to increased numbers of individuals born. All reproductive manipulations entail deviations of natural phenotypes and genotypes, with *in vitro* embryo technologies perhaps showing the biggest effects, although these alterations are still emerging. Most of these indications have been provided by animal models, in particular the bovine species, due to its similarities to human early embryo development. Oocytes and embryos are highly sensitive to environmental stress *in vivo* and *in vitro*. Thus, during *in vitro* culture, a number of stressful conditions affect embryonic quality and viability, inducing subfertility and/or long-term consequences that may reach the offspring. A high proportion of the embryos produced *in vitro* are arrested at a species-specific stage of development during the first cell divisions. These arrested embryos do not show signs of programmed cell death during early cleavage stages. Instead, defective *in vitro* produced embryos would enter a permanent cell cycle arrest compatible with cellular senescence, in which they show active metabolism and high reactive oxygen species levels. Later in development, mainly during the morula and blastocyst stages, apoptosis would mediate the elimination of certain cells, accomplishing both a physiological role in to balancing cell proliferation and death, and a pathological role preventing the transmission of damaged cells with an altered genome. The latter would acquire relevant importance in *in vitro* produced embryos that are submitted to stressful environmental stimuli. In this article, we review the mechanisms mediating apoptosis and senescence during early embryo development, with a focus on *in vitro* produced bovine embryos. Additionally, we shed light on the protective role of senescence and apoptosis to ensure that unhealthy cells and early embryos do not progress in development, avoiding long-term detrimental effects.

## Introduction

Human *in vitro* embryo production has been one of the most remarkable medical achievements of the past century, enabling infertile couples, persons with heritable genetic diseases, or oncology patients to conceive a baby. Since then, more than 8 million babies have been born from assisted reproductive technologies (ARTs) around the world, and it is estimated that around 2.4 million ART cycles are performed every year, leading to 500,000 babies born (Eshre, 2018). The first human baby produced by *in vitro* fertilization (IVF) was born 42 years ago (Steptoe and Edwards, 1978), and since then ARTs have been optimized in various mammalian species. Soon after, IVF was introduced as a very complex experimental procedure in cattle (Brackett et al., 1982), the species other than human with better-developed ARTs. Over the years, the procedures have been simplified and improved due to the development of *in vitro* maturation (IVM) of oocytes recovered from slaughterhouse ovaries or a living animal (transvaginal ovum pickup: OPU), followed by IVF and *in vitro* culture (IVC). Nowadays, *in vitro* embryo production in cattle is not only used to overcome infertility (due to anovulation, fertilization failure (Looney et al., 1994; Block et al., 2010; Gomez et al., 2020), or productive and heat stress (Ealy et al., 1993; Block et al., 2010; Stewart et al., 2011), but as a faster means to obtain embryos of high genetic value. Worldwide, while the number of *in vivo* collected embryos that are transferred seems to have stabilized, the transfer of *in vitro*-produced (IVP) embryos continues to grow (742,908 embryos in 2018) (Viana, 2019). Although there are important differences between cattle and humans that need to be considered, such as the techniques employed, with intracytoplasmic sperm injection (ICSI) being used only in humans, and the age and fertility status of the animals/patients, cattle are frequently used as a model to study preimplantation embryo development, as well as infertility issues in humans, since most technologies used share many similarities. In fact, much of the initial human-based ARTs were based on work completed in cattle (Sirard, 2018). *In vitro* studies in cattle with early IVP and *in vivo* developed (IVD) embryos are normally focused on the period into which the blastocyst remains enclosed in the zona pellucida (Rizos et al., 2017). These blastocysts, which are 7–8 days old, bear the best pregnancy and birth rates for embryo transfer (ET) to recipients (Ledgard et al., 2012; Randi et al., 2016). Subsequent processes (i.e., trophectoderm –TE- elongation in concurrence with gastrulation) are maternally driven in ruminants, with no *in vitro* studies being up to now supportive of a functionally representative elongation.

However, despite all efforts performed to optimize ARTs, gametes and embryos are still submitted to stressful conditions *in vitro*. IVF and intracytoplasmic sperm injection (ICSI) bypass natural selection barriers and involve IVC of gametes and embryos, where nutrients are limited and toxic substrates and end-products of metabolism can be present (Calle et al., 2012; Ramos-Ibeas et al., 2019). Embryos show outstanding plasticity during preimplantation development and can often grow under suboptimal conditions. However, severe alterations can also lead to a state of developmental arrest similar to cellular senescence or trigger apoptotic cell death (Betts and King, 2001). In this review, we shed some light on the debated aspects of embryonic senescence and apoptosis as mechanisms to impede damaged cells and compromised embryos to progress in development. Such important restrictions at peri-conception stages would avoid long-term deleterious effects on the offspring (Barker, 2007).

## Impact of Assisted Reproduction on Embryo Development: Differences Between *in vivo* and *in vitro* Produced Embryos

Despite many improvements made in the field of assisted reproduction, *in vitro* embryo production systems are still not as efficient as *in vivo* development (Rizos et al., 2017). Notwithstanding, it should be emphasized that gametes and embryos are exposed to spatial and temporal unnatural conditions during ART, whose consequences are not completely known (Van Eetvelde et al., 2017). In cattle, approximately 90% of oocytes cultured *in vitro* undergo nuclear and cytoplasmic maturation, from which 80% are fertilized and cleave at least once (Lonergan et al., 2003). Contrary to *in vivo* development, whereby one or less frequently two oocytes are ovulated in the cow, *in vitro* procedures permit maturation of virtually all oocytes in the absence of the inhibitory effects of the dominant follicle (Labrecque et al., 2016; Nagano, 2019). Nonetheless, only between 30 and 40% of such oocytes reach the blastocyst stage (Rizos et al., 2017). While at first appearance this may appear inefficient, it is important to remember that the oocytes used for IVM come from small (2–8 mm) follicles, which would never ovulate *in vivo* and whose fate would be atresia. Moreover, it has been demonstrated that pregnancy rate following transfer of IVP blastocysts in heifers is approximately 40–50% compared to 60–70% for IVD embryos (Hasler et al., 1995; Hoshi, 2003; see review by Hansen, 2020). Therefore, the challenge today is to improve current IVM conditions to obtain more blastocysts and to improve IVC conditions to produce better-quality blastocysts, capable of continuing development and implantation after transfer to recipient and resulting in births of viable and healthy individuals.

Oocyte developmental competence, often defined as the ability of the oocyte to mature, be fertilized, and develop to the blastocyst stage, has been associated with the size of the antral follicle from which it is recovered, the stage of the follicular wave, and the site of maturation—*in vivo* or *in vitro* (Pavlok et al., 1992, see review by Lonergan and Fair, 2016). Oocytes matured *in vivo* are of better quality than those matured *in vitro*, and this is reflected in the rates of embryos produced subsequently. Indeed, irrespective of whether embryo development occurred *in vivo* or *in vitro*, when bovine oocytes were matured *in vivo* from superovulated cows, the resultant blastocyst rate was almost 80%, while when oocytes were matured *in vitro*, it was limited to about 35% (Rizos et al., 2002).

In mammals, the cumulus-oocyte complex (COC) provides metabolites and nutrients, like pyruvate, oxaloacetic acid, and amino acids to the oocytes during their growth, besides stimulating them to resume meiosis and progress to metaphase II (Chen et al., 1990). Several studies demonstrated that gene functions and signaling pathways interact between the oocyte and CCs (Regassa et al., 2011). Thus, gene expression patterns in CCs are currently used as indicators of oocyte quality (Tesfaye et al., 2009; Bunel et al., 2015). Besides, IVM and *in vivo* maturation differ in the amounts of mRNA transcripts stored in the ooplasm (Lonergan et al., 2003), with more than 100 differentially expressed genes between *in vitro*- and *in vivo*-matured bovine oocytes being responsible for depleting their developmental competence (Katz-Jaffe et al., 2009; Adona et al., 2016).

In humans, IVM can be an alternative for patients with ovarian pathologies affecting oocyte quality (Ata et al., 2010; Shalom-Paz et al., 2010) and can also reduce the risks of ovarian hyperstimulation syndrome (Sauerbrun-Cutler et al., 2015). However, despite its potential benefits, IVM is still marginally used in human ART, with applications mainly in fertility preservation (Kasum et al., 2015; Shirasawa and Terada, 2017). Therefore, a better understanding of the mechanisms involved in oocyte competence during IVM is crucial in the optimization of this technology.

The fate of an embryo is determined at fertilization. Delays in fertilization or fertilization by a damaged spermatozoon could conceivably lead to oocyte aging or to the formation of a defective embryo, respectively (Tarín et al., 2000). However, in the bovine species, IVC seems to be one of the major factors determining embryo quality. As mentioned before, IVC embryos show poorer morphology, cryotolerance, distinct transcript expression profiles, and pregnancy rates after transfer than IVD embryos (see review by Lonergan, 2007). Alternating *in vitro* and *in vivo* embryo culture demonstrated the importance of developing *in vitro* systems mimicking the *in vivo* situation. Thus, the culture of IVP bovine zygotes *in vivo* in the oviduct of sheep (Rizos et al., 2002), cow (Tesfaye et al., 2007), or mouse (Rizos et al., 2007), increased the quality of the resulting blastocysts. Conversely, IVC of *in vivo* produced bovine zygotes resulted in blastocysts of low quality (Rizos et al., 2002). However, IVC before and during embryonic genome activation (EGA) did not affect embryo development rates, irrespective of where culture took place, although IVC did affect the transcriptome of such blastocysts (Gad et al., 2012).

Negative factors within IVC include media supplementation with fetal calf serum (FCS), which reduces embryo cryotolerance, induces alterations in gene expression and leads to large offspring syndrome in cattle, a disorder caused by epigenetic alterations in the embryo that is phenotypically similar to Beckwith-Wiedemann syndrome in humans (Young et al., 1998; Lazzari et al., 2002; Rizos et al., 2003; Wrenzycki et al., 2005). Compared to *in vivo* derived embryos, IVP blastocysts produced with FCS had 5 times more differentially expressed genes than serum-free IVP blastocysts (1,109 vs. 207) (Heras et al., 2016). Serum derivatives added to culture can also trigger long term detrimental effects, as shown by improved survival to cryopreservation and reduced miscarriage rates observed when bovine serum albumin (BSA) was removed from the culture at the time of blastocyst formation (Murillo-Rios et al., 2017; Gomez et al., 2020). The embryonic genome is epigenetically reprogrammed after fertilization. This process involves activating or silencing specific genes by laying on appropriate methylation patterns (Reik et al., 2001). During this critical period, the embryo is especially vulnerable to epigenetic defects, which can be induced by IVC (El Hajj and Haaf, 2013). Indeed, animal studies have revealed links between different ARTs and imprinting disorders, via altered DNA methylation patterns and histone codes (Urrego et al., 2014). Such disorders are more prevalent in gametes and embryos after ARTs than in their *in vivo*-derived counterparts (Urrego et al., 2014). Thus, IVP embryos show culture-induced, stage-specific, and non-stage-specific, aberrant DNA methylation patterns in paternally or maternally imprinted genes and in several genomic clusters (Salilew-Wondim et al., 2015, 2018). The post-fertilization culture environment is therefore crucial for the quality of the resulting blastocysts (see review by Rizos et al., 2017; Wrenzycki, 2018). To overcome the limitations of conventional IVC and mimic *in vivo* conditions, different embryo culture systems have been developed such as co-culture with bovine oviduct epithelial cells (BOECs) (Schmaltz-Panneau et al., 2014) or supplementation of culture media with oviductal and uterine fluid in sequential culture, as well as the use of extracellular vesicles (EVs) (Rodriguez-Alonso et al., 2020). All these systems entail improvements in IVC for blastocyst development and their quality in terms of cryotolerance, cell counts, cell apoptosis and gene expression (Alminana et al., 2017; Lopera-Vasquez et al., 2017; Almiñana and Bauersachs, 2019). In particular, EVs secreted by donor oviductal cells increased birth rates after embryo transfer in mice due to decreased apoptosis and improved cellular differentiation in embryos (Qu et al., 2019). Furthermore, supplementation of IVC medium with uterine exosomes improved the developmental capacity of embryos generated by somatic cell nuclear transfer (SCNT) (Qiao et al., 2018). Taking together the above findings, it could be suggested that the effect of EVs on early embryonic development may be fundamental, and IVC systems containing EVs can provide new insights to improve gamete maturation, fertilization, and embryo development during ARTs.

In parallel, other research lines based on chemical modifications of simple medium, polarized and three-dimensional (3D) cell co-cultures, lead to improved embryo development and quality. These systems could allow cultured gametes and embryos to experience some of the same mechanical forces, mimicking interactions with the substratum of the oviduct and endometrium, and dynamic changes that occur in the microenvironment of the reproductive tract. The BOEC polarized system consists of growing the cells on inserts to allow media access from basolateral and apical sides, and to maintain the polarized asymmetrical structure of the oviductal epithelial cells. Epithelial cells derived from human, porcine or bovine oviduct maintained polarity and *in vivo*–like morphology when they were cultured for long term in a polarized air-liquid interface system (Levanon et al., 2010; Chen et al., 2013, 2017). It was evidenced that air-liquid system supports embryo development *in vitro* without culture medium supply in porcine, mouse and bovine species; however, the obtained blastocyst rates could not yet match the outcome of optimized standard IVP procedures, suggesting that further improvement of the model is required (Chen et al., 2017). In cattle, using the 3D printing technology in combination with microfluidics resulted in the creation of “oviduct-on-a-chip” with a U-shaped porous membrane allowing BOEC polarization that could be maintained during long-term mimicking tissue- and organ-specific micro-architecture (Ferraz et al., 2017b). It was demonstrated that culturing BOEC in 3D system improved embryo production by allowing proper sperm and oocyte interactions, fertilization, and completely abolishing polyspermic and parthenogenic activation of oocytes in the absence of added sperm activating factors (Ferraz et al., 2017a). Although technical issues contributed to lower cleavage and developmental rates than in a conventional embryo production system, the DNA methylation intensity and transcript abundance in zygotes produced in the microfluidics device were more similar to embryos produced *in vivo* than to embryos produced in a conventional IVP system (Ferraz et al., 2018).

## Senescence During Early Embryo Development

Cellular senescence is a state of irreversible cell cycle arrest that can be induced through different mechanisms, such as genomic and telomeric damage, epigenetic perturbations, reactive oxygen species (ROS), or activation of oncogenes, and mitogenic signals (Lopez-Otin et al., 2013). This event was first noticed in primary human fibroblasts that had undergone replicative exhaustion after a finite number of passages (“Hayflick’s limit”) (Hayflick and Moorhead, 1961). Nowadays, this state is known as replicative senescence and is associated with telomeres shortening. However, stress-induced premature senescence can be also induced as a defense mechanism in damaged cells that are not able to undergo apoptosis, and programmed senescence has been reported during development (Lozono-Torres et al., 2019).

Senescent cells show specific morphological and biochemical features, often presenting a large flattened morphology with increased cytoplasmic and nuclear volume in culture (Rhinn et al., 2019). These cells remain metabolically active, resist to apoptosis-inducing stimuli (Baar et al., 2017) and show high ROS levels and β-galactosidase activity (Wang and Dreesen, 2018). At the molecular level, the senescent response is mediated by two main components: the intrinsic arm and the extrinsic arm (Rhinn et al., 2019). The intrinsic arm is mediated by proteins that inhibit cell cycle progression, including tumor protein P53 (p53), p21 (encoded by Cyclin Dependent Kinase Inhibitor 1A; *Cdkn1a*), p16^INKA^ and p19^ARF^ (both encoded by Cyclin Dependent Kinase Inhibitor 2A *Cdkn2a*) (Kuilman et al., 2010; Martinez-Zamudio et al., 2017), and by microRNA-mediated gene silencing (Benhamed et al., 2012). The extrinsic arm comprises the “senescence-associated secretory phenotype” (SASP), mediated by cytokines, chemokines, growth factors, extracellular matrix (ECM), and ECM-remodeling proteins (Freund et al., 2010; Acosta et al., 2013) that are transcriptionally regulated by nuclear factor kappa-light-chain-enhancer of activated B cells (Nfkb), CCAAT/enhancer-binding protein beta (Cebpβ), p38, p53, and GATA binding protein 4 (Gata4) transcription factors (Acosta et al., 2008; Kuilman et al., 2008; Kang et al., 2015). The SASP allows senescent cells to secrete signaling molecules, generating a microenvironment, and activating the immune system to remove damaged or stressed cells (Kang et al., 2011; Sagiv et al., 2016).

Although cellular senescence has been mainly associated with tumor suppression, tissue repair or aging (Baker et al., 2011; Campisi, 2013; Ritschka et al., 2017), features of senescence have been also reported in cells from different mammalian species during embryonic development (Rhinn et al., 2019). In the mouse, senescent cells have been identified by expression of β-galactosidase, p21 and SASP factors in many tissues during specific time windows of fetal development (mesonephros, neural tube, gut endoderm, heart, and the developing limbs, etc.) (Munoz-Espin et al., 2013; Storer et al., 2013; Lorda-Diez et al., 2019). These cells emerge and are removed by macrophages in a tightly controlled manner, resulting in tissue remodeling and patterning. Interestingly, senescent cells in the developing fetus are negative to certain senescence markers described in adult tissues, such as p53, p16^INKA^, p19^ARF^, and DNA damage. Instead, developmental senescence is strictly dependent on p21 and is regulated by the TGFβ/SMAD and PI3K/FOXO pathways (Munoz-Espin et al., 2013; Storer et al., 2013; Table 1). This senescence model seems to be conserved in vertebrate development, as similar patterns have been observed in chick (Storer et al., 2013) and human embryos (Munoz-Espin et al., 2013), and could have emerged initially as a basic developmental mechanism that subsequently evolved to a protective role to face aging and tumor suppression in adult tissues.

**TABLE 1:** Senescence and apoptosis markers detected through development.

Senescence	Adult	Fetus	Early embryo
p53	Yes	No	No
p21	Yes	Yes	Yes
p16^INK4A^	Yes	No	No
p19^ARF^	Yes	No	?
SASP markers	Yes	Yes	?
P66Shc	Yes	?	Yes
Phosphorylated γH2AX	Yes	No	Yes
β-galactosidase	Yes	Yes	Yes
APOPTOSIS			
p53/phospo-p53	Yes	No	No
p21	Yes	Yes	Yes
BAX	Yes	Yes	Yes
Bcl-2	Yes	Yes	?
DNA fragmentation	Yes	Yes	Yes
Apo-1/Fas, FasL	Yes	Yes	No
Activated caspases	Yes	Yes	No
PS externalization	Yes	?	Yes

**SASP, Senescence-associated secretory phenotype.**

**FIGURE 1:**
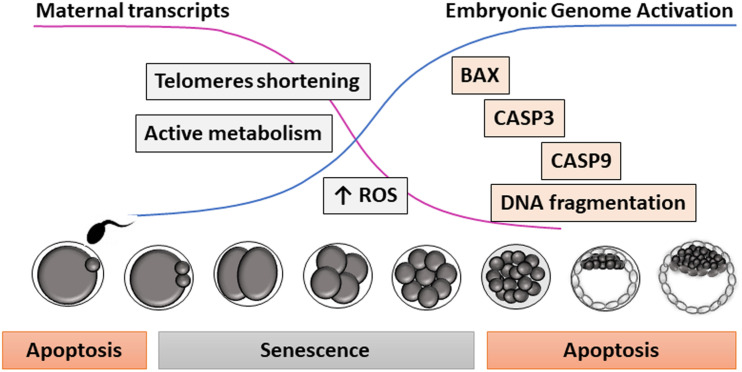
Proposed model for apoptosis and senescence during early embryo development. Due to the low amount of specific embryonic transcripts before embryonic genome activation (i.e., telomerase), embryos are very sensitive to environmental stressors during early cleavage stages, entering into a state of cell arrest resembling senescence. Later in development, embryos (and oocytes before fertilization) can show DNA fragmentation and other morphological signs of apoptosis.

Going one more step back in development up to the preimplantational embryo, the existence of senescence was first hypothesized almost 20 years ago (Betts and King, 2001). A high proportion of the embryos produced *in vitro* are arrested at a species-specific stage of development during the first cell divisions, before EGA. These arrested embryos do not show signs of apoptosis or necrosis, since features of programmed cell death are not detected during the early cleavage stages (Byrne et al., 1999; Hardy, 1999; Matwee et al., 2000). Interestingly, when the apoptotic pathway is partially activated by exposing embryos at early cleavage stages to protein kinase inhibitors or mitochondria depolarizing agents, limited caspase activation, and DNA fragmentation can be observed (Matwee et al., 2000; Gjorret et al., 2007). This might suggest that components of the apoptosis machinery are available before EGA, but cannot be physiologically activated due to the absence of mature mitochondria (Plante and King, 1994; Van Blerkom, 2004) or to certain inhibition of the apoptotic pathway (Brad et al., 2007). Instead, defective IVP embryos would enter a state of permanent cell cycle arrest before EGA compatible with cellular senescence, in which they show active metabolism and high ROS levels (Betts and Madan, 2008; Figure 1).

**FIGURE 2:**
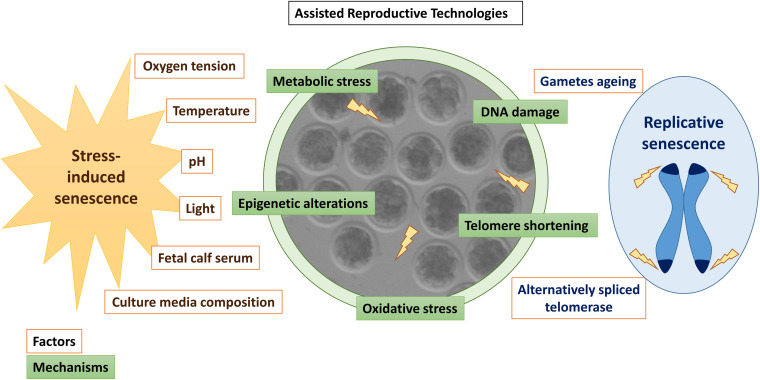
Summary of factors (orange) and mechanisms (green) inducing senescence in *in vitro* produced embryos. Stressors associated with *in vitro* embryo culture are written in brown letters, while factors associated with replicative senescence are written in blue.

Assisted reproductive technologies induce oxidative stress in the embryos (Ramos-Ibeas et al., 2019), which might also generate DNA and telomeric damage before EGA (Betts and Madan, 2008). At these stages of development, many transcripts are scarce, including telomerase, the enzyme responsible for telomere elongation (Betts and King, 1999). Alternatively spliced telomerase variants associated with a lack of telomerase activity have been detected in some human oocytes and poorly developing embryos (Brenner et al., 1999). Moreover, telomere length in human oocytes predicts embryo fragmentation at day 3 of development, supporting the theory of reproductive senescence in women (Keefe et al., 2005). Thus, stress-mediated telomeric damage due to sub-optimal culture conditions, shortened telomeres derived from aged oocytes, or absence of proper telomerase activity in low-quality oocytes or embryos could trigger embryo senescence before EGA (Betts and King, 2001; Figure 2).

In contrast to senescent adult cells and in the same way as in fetal cells, p53 is not involved in embryo senescence during early cleavage stages (Favetta et al., 2004; Favetta et al., 2007a; Velez-Pardo et al., 2007; Meuter et al., 2014). Furthermore, p16^INKA^, which is involved in the senescence response in adult tissues, has not been detected in embryos (Egashira et al., 2011; Meuter et al., 2014). However, IVP bovine embryos arrested at the 2-cell stage were positive for phosphorylated γH2AX, a senescence marker that was not detected in proliferating 2-cell embryos (Betts and Madan, 2008). Besides, a direct correlation between the oxidative stress adaptor protein p66Shc levels and the duration of the embryonic arrest was found in these relatively transcriptionally silent embryos, so p66Shc might play a key role in the senescence response induced by ROS in early embryos (Favetta et al., 2004, 2007b). Additional markers of senescence in IVP embryos are β-galactosidase and p21 expression (Meuter et al., 2014; Ock et al., 2020; Table 1). Upregulation of p21 has been reported in mouse preimplantation embryos in response to heat stress or X-ray radiation that led to developmental delay or blockage in damaged embryos (Ock et al., 2020). In the absence of p21, damaged embryos can progress to the blastocyst stage, although exhibiting increased apoptosis and chromosome instability (Adiga et al., 2007). Thus, p21 plays a protective role in the embryo, mediating a senescent phenotype in response to exogenous stressors.

Therefore, senescence seems to play a protective role during preimplantational development, acting as a checkpoint mechanism to ensure that unhealthy embryos enter into a permanently arrested state and do not progress in development, avoiding long-term effects on health and reproduction. This mechanism would become even more important in IVP embryos, which are exposed to multiple stressors (Ramos-Ibeas et al., 2019). It would be interesting to investigate whether senescent embryos secrete signaling factors that could exert a detrimental effect on the rest of the embryos in culture.

## Apoptosis During Early Embryo Development

Cell death exerts an essential role in governing early mammalian embryo development (Hardy, 1997; Betts and King, 2001; Leidenfrost et al., 2011; Vandaele and Van Soom, 2011). Early embryos progress in development faster than tumor cells because of the lower embryonic demand for membranes construction and lack of new biomass synthesis (Krisher and Prather, 2012). A delicate balance between cell proliferation and death is therefore required for successful development to term. This view of cell death, as a regulatory process, alternates with its pathological role (Betts and King, 2001; Leidenfrost et al., 2011). Regulated cell death (RCD) exists mainly in multicellular organisms as a form of genetically programmed elimination of cells that are perceived as dispensable, noxious, or with irreversible damage. Physiological forms of RCD do not require intracellular or extracellular alterations (Conradt, 2009; Fuchs and Steller, 2011). On the contrary, prolonged and/or intense stimulation, insofar as they produce stress and disturb cellular homeostasis, induce pathological RCD (Galluzzi et al., 2016). Apoptosis is a form of RCD existing in multiple tissues during embryonic and fetal development in mammals. The apoptotic cell is rounded and shrunk, showing condensed, marginalized, and later fragmented chromatin, intact nuclear envelope, shrunk organelles and contained cytoplasm (Voss and Strasser, 2020). A recent hypothesis postulated that apoptosis and cell proliferation are independent processes that work together better than alone in regulating growth (Voss and Strasser, 2020). The role of apoptosis in mammalian development is not only to control cell proliferation, including the deletion of cells and portions of tissues, but also to regulate morphogenesis, including the formation of cavities, and vesicles in compact structures and modeling tissue shape (reviewed by Diamantis et al., 2008). Since the framework of early morphogenetic events is not yet started in early embryo development, the apoptosis mechanisms seem to be limited to eliminate abnormal cells, as there is no information about apoptosis regulating other essential hallmarks until the blastocyst stage. A recent study in a mouse model of chromosome mosaicism supports this hypothesis, where abnormal aneuploid cells are eliminated by autophagy and apoptosis at the peri- and post-implantation stages of development (Singla et al., 2020). In human ART, apoptosis has been postulated as a candidate mechanism to explain elevated monozygotic twining (MZT), which would be facilitated by more labile junctions between the ICM cells in IVP embryos (Hviid et al., 2018). Thus, high glucose in culture increases MZT rates, explained as increased ROS production and subsequent apoptosis that would disrupt the ICM, with the possible help of overpressure during hatching (Ménézo and Sakkas, 2002). Frequent blastocyst collapse could relocate some ICM cells on the trophectoderm wall, thereby forming a second, ectopic ICM, and subsequent MZT (Payne et al., 2007). Embryo biopsy and cryopreservation, together with specific donor conditions have also been cited as factors underlying increased MZT, always with apoptosis as a triggering factor (reviewed by Tauwinklova et al., 2010). In the cow, increased MZT has been not documented within IVP embryos.

Thus, the interest in apoptosis within *in vivo* and *in vitro* produced embryos lies in its strong consistency with a variety of stresses and poor development conditions, and this makes the apoptosis signs timely markers of an abnormal environment and likely responsible of increased MZT in humans. Hence, one of the hallmarks of IVP is an apoptosis rate generally higher than embryos naturally developing in the genital tract (Pomar et al., 2005), indicating that *in vitro* conditions are poorer for the embryo.

### Apoptotic Pathways Within Early Embryos

Apoptosis is regulated through two convergent pathways, depending on whether the signals initiating cell death originate within or outside the cell: (1) the extrinsic or death receptor pathway, mainly activated by members of the tumor necrosis factor (TNF) family that bind to their family of cognate receptors (TNFR), and (2) the intrinsic, mitochondrial, or B-cell lymphoma 2 (BCL-2) regulated pathway. Both apoptotic pathways are active –but not completely- in early embryos under stage-specific conditions (Leidenfrost et al., 2011).

#### Intrinsic Apoptosis

Intrinsic apoptosis pathway proceeds at least by well-defined environmental perturbations (Galluzzi et al., 2018). The bovine embryo has been identified as responsive to some of such alterations: (1) Endoplasmic reticulum (ER) stress, which simultaneously increases expression of ER stress (Glucose-Regulated Protein, 78 kDa (*GRP78*), Activating Transcription Factor 4 (*ATF4*), Activating Transcription Factor 6 (*ATF6*), Regulator Of G Protein Signaling 1 (*RGS1*) and X-Box Binding Protein 1 (*XBP1*)] and pro-apoptotic [C/EBP Homologous Protein (*CHOP*) and BCL2 Associated X, Apoptosis Regulator (BAX)] genes, at the same time that development rates, cryotolerance, cell survival and apoptotic cell rates improve (Yoon et al., 2014; Khatun et al., 2020); (2) ROS overload can also trigger apoptosis and developmental affectance (Huang and Chan, 2017; Luo et al., 2020); (3) Irregular DNA structures, out of which only a subset can be identified by TUNEL (Gjorret et al., 2003; Leidenfrost et al., 2011); abnormal segregation of chromosomes in mitosis is a possible origin of such structures (Gjorret et al., 2003); (4) Mitotic effects, where apoptosis was observed in response to multinucleate cells (Paula and Hansen, 2008) and cytokinesis becomes blocked leading to a “mitotic catastrophe” by checkpoint activation (Huang et al., 2005); (5) Specific miRNAs that trigger apoptosis, since slow-cleaving bovine embryos in culture release miRNA-30c, which targets cyclin-dependent kinase 12 (CDK12) and lead to apoptosis (Juan et al., 2016; Lin et al., 2019), although it is unknown whether dead cells or live cells with a commitment release miR-30c; 6) Growth factors removal as shown by specific growth factors identified in the genital tract that reduce apoptosis when added to IVC (Trigal et al., 2011; Gomez et al., 2014). In pigs, vascular endothelial growth factor (VEGF) decreased in parallel to an increase in embryo development, concomitant with a reduction in mRNA abundance of caspase 3 (*CASP3*) and increased BCL2 Apoptosis Regulator (*BCL2*) and Nuclear Factor, Erythroid 2 Like 2 *NRF*-2 (Biswas et al., 2018).

Synergies between the above inducers may exert a more tuned regulation of apoptosis and proliferation, as shown in cancer cells, where more than 300 miRNAs respond to retinoic acid (RA) to inhibit cell invasiveness and deregulate growth (Lima et al., 2019). Interestingly, RA is a main activated pathway in the bovine uterus (Bauersachs et al., 2005). *In vitro*, RA regulates apoptosis in the bovine blastocyst through both retinoid receptors RXR and RAR (Rodriguez et al., 2006, 2007; Gomez et al., 2008).

#### Extrinsic Apoptosis

Extracellular perturbations can trigger apoptosis through the extrinsic pathway, activated by receptor-ligand interaction between extrinsic molecules and mainly two types of receptors in the plasma membrane: (1) Death receptors, activated by cognate ligands; (2) Dependence receptors, activated by falling of ligand concentrations below specific thresholds (Galluzzi et al., 2018). Death receptors include Fas cell surface receptor (FAS, also termed as APO-1 or CD95), and TNF receptor superfamily member 1A (TNFR1), 10a (DR4 or TRAILR1), and 10b (DR5 or TRAIL2) (Wajant, 2002; Aggarwal et al., 2012; Von Karstedt et al., 2017). The extrinsic apoptosis pathway could be partly inactive in bovine embryos because of the lack of convergence with the intrinsic pathway to activate the effector caspases, since caspase-8 would be absent or inactive. Together with caspase-8, FAS/FASL transcripts are at very low abundance or absent in embryos (Leidenfrost et al., 2011). Furthermore, the FAS signaling pathway is inactive in bovine, human and mouse oocytes (Sato, 1998; De Los Santos et al., 2000; Pomar et al., 2004). The alternative pathway of necroptosis, a regulated common morphology of necrotic and apoptotic cells, which in turn should become active by caspase-8 inhibition, has not been studied in bovine embryos to our knowledge. However, morphological evidence associated with this RCD type has been described (Gjorret et al., 2003; Rodriguez et al., 2006). A third signaling pathway through death receptor is represented by Nuclear factor-Kappa-B (NF-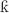
h) activation, which usually leads to a wide inflammatory response linked to cell survival (Nguyen-Chi et al., 2017; Von Karstedt et al., 2017). The bovine blastocyst is equipped with NF-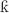
B inducers, such as TNF (Correia-Alvarez et al., 2015b) and Interleukin 1 Beta (IL1b) (Correia-Alvarez et al., 2015a). In the uterus, Day 6/Day 8 embryos need to depress a natural pro-inflammatory status to progress in development; evidence of such depression, including reduced NF-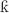 in the uterine fluid, has been provided (Munoz et al., 2012). Interestingly, the sex of the embryo determines the extent of such a response in the uterus, with female blastocysts showing higher apoptotic rates in their ICM (Gomez et al., 2013).

### Apoptosis Chronology During Early Development Stages

The timing at which the IVP embryo responds to external stimuli through apoptosis -as an endogenous mechanism of removing cells- has been defined in stage-specific studies with embryos. However, perturbations of the oocyte and spermatozoa environments can also result in an apoptotic response in the generated embryo (El Hajj and Haaf, 2013; Wang et al., 2017; Bittner et al., 2018), although this topic is out of the scope of the present review.

Apoptosis and kinetic patterns of development are related, since the incidence of embryos with dying/dead cells increases through development by stage and day. However, the most advanced embryonic stages within a day show lower apoptosis rates. Pioneering studies (Byrne et al., 1999; Matwee et al., 2000; Gjorret et al., 2003) defined the chronology of apoptosis within IVD and IVP cattle embryos. Embryos were examined by morphological apoptotic traits (M) and analysis of DNA fragmentation using terminal deoxynucleotidyl transferase-mediated dUTP nick end labeling (TUNEL) (T) (Gjorret et al., 2003). Only nuclei showing T + M were regarded as completely apoptotic. Apoptotic M was rarely detectable before the 9–16 cell stage. In contrast, significant proportions of mature and immature oocytes showed T staining (Matwee et al., 2000), indicating that apoptotic processes became repressed after fertilization, since no IVD embryos and very few IVP embryos (< 3%) showed complete apoptotic T + M up to the morula stage. The first sign of T + M was observed at the 8-cell stage (Byrne et al., 1999; Matwee et al., 2000; Paula-Lopes and Hansen, 2002; Gjorret et al., 2003), while close to 50% Day-3 IVP embryos (i.e., at different development stages) contained at least one cell showing any sign of M (Leidenfrost et al., 2011). Thus, at the blastocyst stage rarely an embryo was found without dying/dead cells, with steadily higher apoptotic rates in the ICM (usually 25–40% total cells in blastocysts) than in the trophectoderm (Gjorret et al., 2003; Rodriguez et al., 2006; Gomez et al., 2008, 2014, 2020; Leidenfrost et al., 2011; Munoz et al., 2014; Murillo et al., 2017), suggesting a more stringent regulation in the developmentally important and yet undifferentiated ICM. Such differences are not apparent in human embryos, although appropriate studies are limited and with low sample numbers (Sjöblom et al., 2002; Hardy et al., 2003).

**FIGURE 3:**
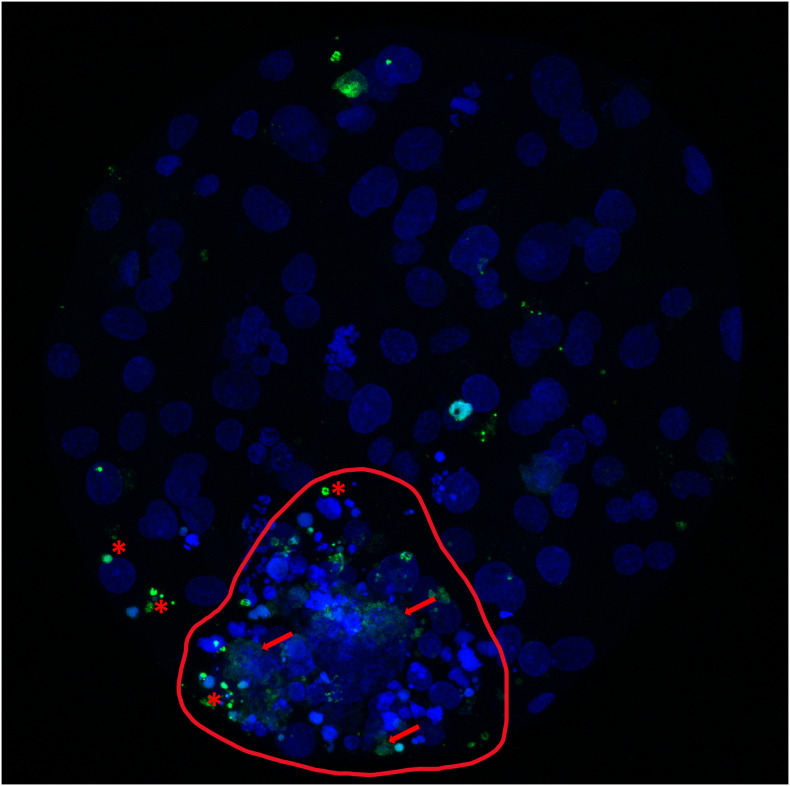
Cell death analysis in a bovine expanded blastocyst produced *in vitro*. Bovine expanded blastocyst produced *in vitro* and stained with bisbenzimide (blue) to visualize all nuclei and TdT-mediated dUTP nick-end labeling (TUNEL; green). Red asterisks mark TUNEL-positive nuclei undergoing the apoptotic pathway (condensed, TUNEL-positive structures that can appear scattered as smaller apoptotic bodies and correspond to chromatin and cytoplasmic condensation, with intact nuclear membrane). Red arrows mark nuclei undergoing necrosis (TUNEL-positive structures, with unclear, swollen and frayed edges; red arrows). The inner cell mass (circled, red line) shows the highest incidence of identified and non-recognizable forms of cell death, in contrast with a lower incidence in the more abundant trophectoderm cells.

Signaling pathways and genes involved in the apoptotic response have been analyzed during early embryo development in the bovine species. In an elegant study, Leidenfrost and co-workers analyzed genes involved in both extrinsic and intrinsic apoptosis initiation (Figure 3; Leidenfrost et al., 2011). Within IVP embryos, the copy numbers of *CASP3*, X-linked Inhibitor of Apoptosis (*XIAP)*, *BAX*, BCL2 Like 1 (*BCL2L1)*, Caspase 9 (*CASP9)*, and Signal Transducer and Activator of Transcription 3 (*STAT3)* declined from the oocyte until day 3/4, to gradually increase thereafter. Interestingly, transcripts for the extrinsic initiator caspase-8 were undetectable from the oocyte up to the hatching blastocyst stage (Leidenfrost et al., 2011). Contrary to the caspase-9 protein from the intrinsic apoptotic pathway, caspase-8 was not induced in 2-cell embryos by the apoptotic promoter ceramide, which increases the numbers of multinucleated cells (Paula and Hansen, 2008), an effect by which cytokinesis becomes blocked leading to a “mitotic catastrophe” by checkpoint activation (Huang et al., 2005).

Immunocytochemical staining of the effector protein CASP3, revealed also increasing apoptotic rates from cleavage through the blastocyst stages, but CASP3 positive embryos appeared before the 8-cell stage (Oliveira et al., 2016) at higher rates than those with T + M (see the above-cited works). Such differences may represent incomplete repression of apoptotic mechanisms, or activation of specific subsections, as suggested (Gjorret et al., 2003). The EGA, at the 8–16 cell stage in cattle, entails a complete re-organization of the apoptotic machinery in the embryo, which in this way would reach complete capacity to reorganize cell growth and differentiation. Altered EGA within IVP embryos would be involved in the distinct occurrence of apoptosis features between IVP embryos and their *in vivo* counterparts.

### Embryo Apoptotic Responses to *in vitro* and *in vivo* Stress Sources

**FIGURE 4:**
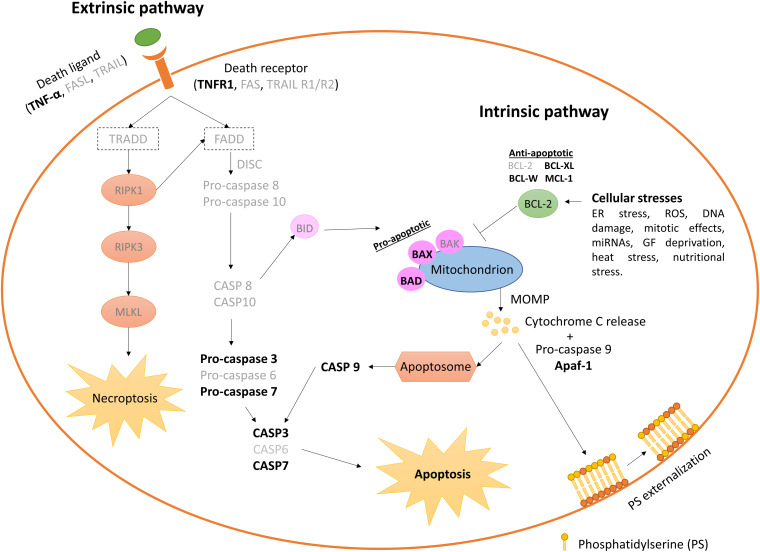
Extrinsic and intrinsic pathways leading apoptosis on the *in vitro* produced bovine embryos. Elements not yet identified in early bovine embryos are shown in lighter types. Only stress sources to which the bovine embryo responds triggering apoptosis cascade are cited. Anti-apoptotic *BCL2* and *BCL-W* gene expression decreases between the oocyte and > 16 cell-stage embryos, while pro-apoptotic *BAX* increases. *BAX* and *BCL2* are absent in > 20 cells embryos (Melka et al., 2010; Fear and Hansen, 2011), although other authors have reported absence of BCL2 during early embryo development (Leidenfrost et al., 2011). CASP3 and CASP9 are found at very low abundance, and CASP8 is undetectable neither in oocytes nor in embryos at any analyzed stage (Leidenfrost et al., 2011). DISC, death-inducing signaling complex; MOMP, mitochondrial outer membrane permeabilization.

Early embryos can respond to a variety of stressors through apoptosis activation *in vivo* and *in vitro* (Figure 4). Heat-shock-induced apoptosis can occur in cattle embryos exposed to high environmental temperatures, being more acute in high-producing dairy cows due to their higher metabolic heat; 8–16 cell embryos are the first stage able to respond to heat stress (De Barros and Paula-Lopes, 2018). Nutritional stress is also harmful for embryo development and pregnancy. In a diabetic pregnancy, hyperglycemia has been related with congenital malformations in humans and animals (Eriksson and Wentzel, 2016; Shub and Lappas, 2020). Mimicking maternal diabetes with very high concentrations of glucose in culture reduces blastocyst development rates and trophectoderm cells, while increases apoptotic cell rates in bovine and mouse (Bermejo-Alvarez et al., 2012; Uhde et al., 2018). The metabolomic profile of bovine embryos produced in high-glucose medium showed an increase in glycolysis and hexosamine pathways, and a reduction in the components of the tricarboxylic acid cycle, resulting in a deficient energy production from pyruvate, and in an increase in the polyol pathway as an alternative to produce energy (Uhde et al., 2018). Also, high glucose increased the expression of pro-apoptotic genes *Bax* and *Casp3* and decreased the mitochondrial content and expression of glucose transporters in mouse blastocysts (Shen et al., 2009). All these alterations may induce oxidative stress and the activation of the apoptosis pathway in the embryo (Jimenez et al., 2003). Moreover, negative energy balance (NEB) in cows and obesity in humans are associated with upregulated lipolysis, which increases non-esterified fatty acids (FA) concentrations in blood. Bovine blastocyst obtained from COCs matured with a high concentration of FA had an increased apoptotic incidence and lower cell numbers (Van Hoeck et al., 2011; Marei et al., 2017), caused by lipid accumulation and increased mitochondrial ROS (Prastowo et al., 2016).

Incidence of apoptosis within *in vivo* embryos is generally lower than in IVP embryos (Gjorret et al., 2003; Pomar et al., 2005; Warzych et al., 2007), although this appreciation is not unanimous (Leidenfrost et al., 2011). Thus, embryonic quality is normally associated with lower apoptotic rates. IVP blastocysts show higher transcript levels for *XIAP*, *BAX*, *BCL2L1*, *CASP3*, and *CASP9* than *in vivo* embryos. However, neither *in vivo* nor IVP embryos show transcripts for *BCL2*, Caspase 8 (*CASP8)*, and Fas Ligand (*FASLG)*; and Fas Cell Surface Death Receptor (*FAS)* transcripts were detected only in the *in vivo* group at very low abundance (Leidenfrost et al., 2011).

Supplementation of *in vitro* culture media with serum increases embryonic Bax expression (Rizos et al., 2003), while improved formulations that increase the stability of glutamine (e.g., as glutamyl-glycine), avoids ammonia production by glutamine degradation (Zuo et al., 2020). Embryo cryopreservation, in particular vitrification and warming, enhances the expression of apoptotic genes like Forkhead Box O3A (*FOXO3A)*, Patatin Like Phospholipase Domain Containing 2 (*PNPLA2*), *BCL2L1*, and *BAX*, although apoptotic phenotypes do not necessarily appear modified (Madrid Gaviria et al., 2019).

Interestingly, Day-6 IVP female embryos can show higher apoptotic rates than males (Oliveira et al., 2016). Such differences, nevertheless, can be due to culture conditions potentially inappropriate for female embryos, as there is sufficient evidence of metabolic differences between males and females (Sturmey et al., 2010; Munoz et al., 2020) and no sex-specific culture media have been presently developed. Finally, the individual bull sperm used for IVF can interact with sex factors to alter the incidence of apoptosis during embryonic development (Oliveira et al., 2016).

## Assessment of Embryo Quality Through Senescence and Apoptosis Markers

Until now, the most widely used method to select IVP embryos for transfer is a morphological evaluation by standard microscopy. However, even when performed by experienced embryologists, it does not completely reflect embryo competence. Cytoplasmic fragmentation and cytokinesis failure, often observed in IVP embryos (Mateusen et al., 2005), can be estimated by morphological evaluation to assess the viability of the embryo, although the outcome can be imprecise and subjective, depending on the evaluator’s criteria. Therefore, the use of alternative markers could improve the selection of more competent embryos. Fragmentation has been associated with apoptosis induction and can lead to a reduction of embryo viability if present to a high degree (Jurisicova et al., 1996). However, although apoptosis evaluation has been suggested as an additional technique to assess embryo quality and viability (Pomar et al., 2005), activation of apoptosis might happen in the embryo as a physiological mechanism to maintain the equilibrium between cell proliferation and death, regardless of external damage or initial oocyte quality (Bakri et al., 2016). In the authors’ opinion, current systems to evaluate apoptosis are relative and invasive. Therefore, due to the large variety of embryo production systems available, measuring a critical level of apoptosis able to trigger developmental arrest is not possible yet, although it would be a highly desirable test to analyze embryonic quality. Comparison between embryo culture systems and *in vivo* models is the reference used in practice. Moreover, the early mammalian embryo is able to cope with a number of adverse environmental situations (Laguna-Barraza et al., 2012; Eckert et al., 2015; Fleming et al., 2015; Hansen et al., 2016) without a clear measurable evidence of the embryo resilience. Thus, appropriate analysis of the efficiency of embryonic “repair mechanisms” (i.e., confident evaluation of embryonic quality) would request pregnancy establishment and progression until birth, which makes such experiments highly expensive or, in most cases, directly unapproachable.

Nowadays, apoptosis can be analyzed by different techniques, differentiating those based on biochemical analyses, being the most used the comet and TUNEL reaction assays and annexin V staining, and those based on the analysis of genes and proteins involved in apoptosis pathways. The comet assay is a sensitive technique to detect DNA damage used in numerous eukaryotic cell types and commonly applied in genetic toxicity studies. It is based on electrophoresis migration of denatured DNA fragments and visualization by fluorescence of the comet-like shape that produces the fragmented DNA (Kucharova et al., 2019). Comet is used to detect presence of apoptosis through the visualization of the comet containing broken DNA fragments, but apoptosis level is difficult to quantify. Although in the reproduction field, this technique is mainly used to assess sperm quality and sometimes in oocytes (Chang et al., 2019; Haddock et al., 2020), it has also been adapted to study early embryos, even though the number of studies are very limited and comprise different species. One of its applications is the evaluation of the persistence of induced damage from maternal germ cells in early embryos (Rolland et al., 2017). However, an important limitation of this technique is that it cannot distinguish between DNA fragmentation coming from polar body disintegration (Tatemoto et al., 2000) and DNA fragmentation induced through apoptosis in embryonic cells (Fabian et al., 2003). Moreover, the conventional comet assay cannot distinguish between apoptosis and necrosis, requiring additional techniques to discriminate between them (Morley et al., 2006).

TUNEL assay recognizes fragmented DNA, an event that occurs at the later stage of the apoptotic process. This technique is widely used in studies for quality assessment of embryos and has revealed differences in the onset of apoptosis between *in vitro*- and *in vivo*-produced bovine embryos (Gjorret et al., 2003). Sudano et al. (2014) reported a correlation in apoptosis levels quantified by TUNEL between fresh bovine embryos and post-vitrification survival, which is also used as predictor of embryo quality. In this context, apoptosis incidence has also been analyzed to compare culture medium formulations and IVC conditions, providing information about embryo status. Thus, high oxygen tension and FCS in culture increase apoptosis rates in embryos (He et al., 2020). The effect of FCS is dose-dependent, and very low doses of serum (0.1% FCS) are not pro-apoptotic, although the embryonic quality and competence are reduced (Murillo et al., 2017; Gomez et al., 2020).

Suboptimal conditions during ARTs subject the embryos to chemical and physical stress, triggering apoptosis, and compromising the integrity of the embryo or leading to long-term deleterious effects (Ramos-Ibeas et al., 2019). IVP equine, porcine, ovine, caprine, and bovine embryos show higher incidence of TUNEL quantified apoptosis than their *in vivo* counterparts (Pomar et al., 2005). However, although TUNEL is a frequently used method for apoptosis detection, its specificity is low since the assay labels all free 3/hydroxyl termini in the DNA, which are also detected during necrosis, DNA repair, mechanical damage, and even active gene transcription. Thus, TUNEL staining would detect any type of DNA damage and should be used in combination with other apoptosis-specific assays (Loo, 2011) or analyzing the morphological features of apoptosis and necrosis observed in the target cells (Rodriguez et al., 2006).

Another technique commonly used for apoptosis assessment within IVP is annexin V staining, which allows the detection of phosphatidylserine (PS) in the membrane lipid bilayer surface. The externalization of PS is one of the earliest events in the apoptosis pathway. Annexin V staining has been observed in all human embryos arrested from the 2-cell stage to uncompacted morula, while only 30% of these embryos were TUNEL-positive (Levy et al., 1998), as well as in 1-cell to 9-cell fragmented embryos (Liu et al., 2000). The presence of PS on the outer leaflet of the membrane lipid bilayer of a cell is the earliest event of the apoptosis process. In healthy cells, PS is found exclusively on the inner layer of the membrane cell (Hanshaw et al., 2005). The externalization of PS had been observed by Levy et al. (1998) and Mateusen et al. (2005) in all stages of the apoptotic preimplantation embryo (2-cell stage embryo until blastocyst stage). There are three possible explanations to these results: (1) Annexin V allows the detection of apoptotic cells in arrested embryos earlier than using TUNEL; (2) Annexin V could be also labeling late-stage senescent cells in arrested embryos, which show mitochondrial depolarization by ROS, an event that may precede apoptosis (Wang et al., 2016); and (3) Necrotic cells can exhibit annexin V (Crowley et al., 2016). Therefore, as recommended for TUNEL, detection of annexin V should be combined with other apoptosis-specific assays or apoptotic morphology studies.

Increased expression of pro-apoptotic genes *BAX* and *CASPASE-3* is associated to IVP 2–4 cell bovine embryos bearing poor morphology (Melka et al., 2010). In the same way, the anti-apoptotic gene *BCL2* increases in bovine embryos with good morphological quality (Yang and Rajamahendran, 2002), and in non-fragmented mouse blastocysts (Exley et al., 1999). Therefore, the ratio between pro-apoptotic BAX and anti-apoptotic BCL2 is used to determine embryo quality and marker of differences between IVP and *in vivo* collected embryos. This occurs in bovine (Gutierrez-Adan et al., 2004) and humans (Metcalfe et al., 2004).

MicroRNAs (miRNAs) are key regulators of gene expression through post-transcriptional and post-translational modifications of mRNA. Mammalian blastocysts release miRNA via exosomes and apoptotic bodies in the spent embryo culture medium. Recently, a new approach to study apoptosis through miRNAs in the spent culture medium of individually cultured blastocysts has been proposed. In this study, miR-294 levels in the spent culture medium were directly correlated with apoptosis in mouse embryos. Although the biological role of miR-294 remains unknown, two hypotheses could explain these results. One is that high levels of miR-294 are involved in the suppression of the apoptosis inhibitor *BCL2*, which in the last term acts promoting apoptosis; although no *BCL2* expression could be detected in this study, so this hypothesis could not be confirmed. The other possibility is that apoptosis triggers miR-294 release; which was confirmed by exposing the embryos to ultraviolet radiation (Makri et al., 2020). In bovine embryos, high levels of miR-30c have been associated with increased apoptosis and reduced development rates (Lin et al., 2019). miR-30c, as miR-30 family member, regulates p53 expression and cell apoptosis (Li et al., 2010), and downregulates Cyclin Dependent Kinase 12 (*CDK12)* and DNA damage response (DDR) genes, which may impact on cell cycle progression. miR-30c is highly conserved between species, and in humans, miR-30c levels increase in the spent culture medium of those embryos that will successfully implant (Capalbo et al., 2016). Thus, miRNAs secreted to the culture medium are potential biomarkers of embryo apoptosis and viability, although their biological functions remain unknown or poorly studied. The analysis of compounds secreted to or taken up from the culture media by embryos, such as metabolites and non-coding RNAs, is advantageous, and non-invasive. In contrast, gene expression analysis, TUNEL assay and annexin V staining are invasive techniques that require biopsy or embryo destruction. Biopsy can be detrimental for the embryo and can also lead to false negatives, discarding embryos with actual pregnancy potential.

Controversies between the association of apoptosis and embryo viability still exist (Haouzi and Hamamah, 2009), as shown by similar apoptotic indexes found by TUNEL between developmentally arrested *in vivo* bovine embryos, fragmented morulae and non-fragmented morulae (Ikeda et al., 2006). Similar results have been reported in the human species by TUNEL and Annexin V (Antczak and Van Blerkom, 1999). These results suggest that an alternative process might be going on in poor-quality embryos at these stages, which causes developmental arrest and cannot be detected with the TUNEL assay, such as cellular senescence.

Embryo senescence might underlie the high frequency of early developmental failure associated with sub-optimal culture conditions (Betts and King, 2001). Senescence markers β-galactosidase, phosphorylated H2A.X and p21 (*Cdkn1a*) mRNA are more abundant in mouse IVP than *in vivo* blastocysts. However, their expression was reduced when embryos were cultured in low oxygen tension (5%), pointing to a stress-induced senescence associated with *in vitro* conditions (Meuter et al., 2014). Another marker of embryo senescence could be telomere length in the oocyte, which is negatively correlated with Day-3 human embryos fragmentation (Keefe et al., 2005). However, the use of such senescence markers entails embryo biopsy or destruction to perform Fluorescent *In Situ* Hybridization (FISH), gene expression analysis or embryo staining. Therefore, novel markers and non-invasive methods to predict embryo senescence are still needed.

## Conclusion and Future Directions

Early embryo development represents a very delicate developmental window in which aberrant environmental conditions can easily lead to developmental failure or to long-term negative consequences in health. Due to the increasing use of reproductive technologies in cattle and to the similarities between bovine and human pre-implantation embryos, the bovine species has allowed us to explore many mechanisms involved in developmental failure during this period. However, despite the significant progress made in the field of assisted reproduction during the last decades, still more than 50% of IVP embryos do not reach the blastocyst stage. A high proportion of these embryos enter a state of permanent cell cycle arrest compatible with cellular senescence during the first cell divisions, not showing signs of apoptosis. This protective mechanism might prevent further development of abnormal embryos showing chromosomal anomalies (Almeida and Bolton, 1998) or failure to activate the embryonic genome (Artley et al., 1992), avoiding long-term effects on health and reproduction.

Later in development, after EGA, and mainly at the morula and blastocyst stages, apoptosis would mediate a controlled elimination of certain cells. Apoptosis would then fulfill two different roles: a physiological role maintaining an equilibrium between cell proliferation and death, and a prophylactic role preventing the transmission of damaged cells with an altered genome. This last role would acquire relevant importance in IVP embryos that are submitted to environmental stress, in which apoptosis incidence increases in comparison to IVD embryos. Thus, although later in development, an individual cell might be faced with apoptosis and senescence as alternative fates (Childs et al., 2014), senescence and apoptosis during pre-implantation development would be available during restricted time windows, acting as complementary processes to ensure that damaged cells/embryos do not progress in development.

Currently, common methods to assess apoptosis and senescence in the early embryo, such as immunocytochemistry, gene expression analyses or TUNEL and annexin V staining, are not adequate to evaluate the viability of the embryo before transfer due to their invasive nature. The identification of novel non-invasive markers for apoptosis or senescence remains a crucial goal, such as the analysis of compounds secreted to or taken up from the culture media by embryos, or the possibility of using cumulus cells to analyze the quality of the future embryo, since apoptotic signaling might be already triggered in the oocyte. In parallel, there are other RCD types, such as necroptosis, ferroptosis or cell death induced by the panoptosome, which remain to be explored in early embryos. We hypothesize that such RCD types can be susceptible to alteration in the context of specific traits of early embryonic cells and/or embryo cryopreservation.

Necroptosis is a type of RCD that can be activated through the death receptor pathway via FAS receptor and TNFR1 when caspase-8 lacks or becomes inactive (Shan et al., 2018). In this situation, receptor-interacting serine/threonine-protein kinase-1 (RIPK1) and -3 (RIPK3) activate a mixed lineage kinase domain-like pseudokinase (MLKL). At least in specific circumstances, RIPK1 could also behave as an inhibitor for RIPK3-dependent necroptosis and/or CASP8-dependent extrinsic apoptosis (Orozco et al., 2014). Elucidating these roles in bovine embryo development is challenging. Although necroptosis has not been specifically studied in the early embryo, studies performed by TUNEL alone could have identified a regulated common morphology of necrotic and apoptotic cells. Combining TUNEL staining with a caspase staining may allow such distinction.

Ferroptosis is also a form of RCD driven by iron-dependent lipid peroxidation. Peroxidation of phospholipids, which are integrated into cell membranes, triggers ferroptotic death (Stockwell et al., 2017). The oocyte could be an appropriate objective for ferroptosis damage because of its largest size among the body cells and the high mitochondrial endowment (100,000–300,000 mitochondria in the human gamete (Barritt et al., 2002; Cummins, 2004), which entails a considerable amount and extent of membranes containing the ferroptosis-target phospholipid able to be peroxidized. The mitochondrial numbers decrease with cleavage stages, but they remain higher than normal cells and restart replication in the human expanded blastocyst to reach the maximal counts (Hashimoto et al., 2017) comparable to adult somatic cells (between 80 and 2000 mitochondria per cell) (Cole, 2016). The expanded IVP blastocyst is a preferential stage for cryopreservation, and a ferroptosis target because of the high amount of mitochondrial membranes that can be damaged during cryopreservation.

Panoptosis has been recently described as a multifaceted cell-death signaling platform that integrates multiple cell death types as apoptosis, pyroptosis, and necroptosis which form the collective cell death pathway or PANoptosis (Samir et al., 2020). The PANoptosome drives an inflammatory form of cell death for immune-mediated protection. Modulation of inflammation and immune responses has been described as a strong need in the bovine uterus for embryos to progress in pregnancy (Munoz et al., 2012; Gomez et al., 2013), by which PANoptosis should be considered in future studies that lead to pregnancy establishment in cattle ET.

## Author Contributions

PR-I, IG, KC-B, AG-A, DR, and EG conducted literature review and wrote the manuscript. All authors contributed to the article and approved the submitted version.

## Conflict of Interest

The authors declare that the research was conducted in the absence of any commercial or financial relationships that could be construed as a potential conflict of interest.
